# Leptin to adiponectin ratio in puberty is associated with bone mineral density in 18-year-old males

**DOI:** 10.1016/j.bonr.2021.101158

**Published:** 2021-12-13

**Authors:** Reeli Tamme, Jaak Jürimäe, Evelin Mäestu, Liina Remmel, Priit Purge, Eva Mengel, Vallo Tillmann

**Affiliations:** a Institute of Clinical Medicine, Faculty of Medicine, University of Tartu, Tartu, Estonia; b Institute of Sports Sciences and Physiotherapy, Faculty of Medicine, University of Tartu, Tartu, Estonia; c Children's Clinic of Tartu University Hospital, Tartu, Estonia

**Keywords:** Bone mineral density, Bone mineral apparent density, Leptin to adiponectin ratio, Puberty, Adolescence

## Abstract

**Introduction:**

Inconsistent associations of leptin and adiponectin with bone mineral characteristics in puberty and adolescence have been reported. We aimed to examine the associations between leptin to adiponectin ratio (LAR) in puberty and bone mineral characteristics at the age of 18 years in healthy males.

**Materials and methods:**

88 white Caucasian boys were investigated at T1 (mean age 12.1 years), T2 (14.0 years) and T3 (18.0 years). Serum leptin and adiponectin were measured and LAR was calculated at T1, T2 and T3, bone mineral density (BMD) and bone mineral apparent density (BMAD) for total body and lumbar spine (LS) at T1 and T3. Spearman correlation coefficient and partial correlation analyses were used to describe the associations between mean pubertal LAR and BMD at T3.

**Results:**

Mean pubertal LAR was negatively correlated with both LS BMD (r = −0.23; P < 0.05) and LS BMAD at T3 (r = −0.33; P < 0.05). These associations remained significant also in partial correlation analysis after controlling for total body fat percentage, total testosterone, HOMA-IR and physical activity at T1 (r = −0.31; P < 0.05 and r = −0.41; P < 0.05 respectively).

**Conclusion:**

LAR in puberty is negatively associated with lumbar spine BMD and lumbar spine BMAD at the age of 18 years.

## 1 Introduction

Puberty has a fundamental role in skeletal growth and the accumulation of bone mass (Bachrach, 2001). Peak bone mass obtained during growth and maturation is an important determinant of skeletal health in adult years (Baxter-Jones et al., 2011). Among other factors interfering with bone mass accumulation, adipose tissue-derived adipokines including leptin and adiponectin have been shown to be capable of modulating bone metabolism (Handschin et al., 2007; Scariano et al., 2003; Zhang et al., 2012, Kirk et al., 2020, Fintini et al., 2020, Epsley et al., 2021). Leptin promotes satiety and energy expenditure (Henry et al., 1999), but is also suggested to influence bone tissue formation in humans by enhancing the differentiation of bone marrow stroma cells into mature osteoblasts and inhibiting the differentiation of osteoclasts (Handschin et al., 2007; Scariano et al., 2003). Adiponectin is a protein that is secreted exclusively by adipose tissue and improves insulin sensitivity, thereby regulating lipid and glucose metabolism (Whitehead et al., 2006). Adiponectin has shown direct and indirect effects on bone metabolism through the modulation of several signal transductions and molecules of bone remodelling (Zhang et al., 2012). However, population studies have demonstrated varying relationships between bone mineral density (BMD) and adipokines including adiponectin and leptin (Barbour et al., 2012, Barbour et al., 2011; Haam et al., 2017; Jürimäe et al., 2009, Jürimäe et al., 2008; Lim et al., 2016).

To date, the association between adiponectin and bone mineral variables in children has been studied more in girls and the results are conflicting (Huang et al., 2004; Misra et al., 2007; Rhie et al., 2010). Even more, it has been suggested that the relationship between adiponectin and BMD is sex-dependent as being present in women and not in men (Bi et al., 2020). In our previous cross-sectional study, leptin and adiponectin were not significantly correlated to bone mineral characteristics in boys in early puberty (Vaitkeviciute et al., 2016a), but the longitudinal study showed that leptin, but not adiponectin, at the beginning of puberty, was inversely associated with the following BMD increment over the 24-month period (Vaitkeviciute et al., 2016b). Circulating adiponectin at the age of 10 years was found to be inversely associated with cortical bone mineral content (BMC) and cortical bone area measured by tibial quantitative computed tomography (pQCT) at the age of 15 years (Sayers et al., 2010). To the best of our knowledge, longitudinal relationships between leptin and adiponectin in puberty and bone mineral characteristics later in adolescence have not been studied previously.

Obesity-associated alterations in leptin and adiponectin are major contributors in the development of dysfunctional adipose tissue, characterised by unresolved inflammation (Crewe et al., 2017). Adiponectin to leptin ratio has been used as a marker of dysfunctional adipose tissue (Frühbeck et al., 2017). The leptin to adiponectin ratio (LAR), which has an inversely proportional relationship with adiponectin to leptin ratio, has been shown to correlate with carotid intima-media thickness (Satoh-Asahara et al., 2004) as well as insulin sensitivity (Finucane et al., 2009) and to predict the presence of the metabolic syndrome (Zhuo et al., 2009). Moreover, in adult populations the ratio has proven a more accurate marker of obesity-related complications than adiponectin or leptin alone (Inoue et al., 2006; Zhuo et al., 2009).

Given the critical role of puberty and adipokines in bone mass accumulation, our objective was to study possible associations between LAR in puberty and bone mineral characteristics at 18 years of age in healthy boys.

## 2 Methods

### 2.1 Subjects and study design

The study conducted in 2017–2018 was a follow-up of a longitudinal research project carried out between 2009 and 2013. The baseline data collection was performed in 2010–2011. The invitation to participate in the current study was sent to all 217 boys who were previously recruited from local schools and had participated in the 12-month and 24-month follow-up in 2011–2013. The boys were healthy and had no chronic illnesses. Of these, 104 subjects agreed to participate in the current follow-up study. However, the data from the previous study carried out between 2009 and 2013 were incomplete in 16 subjects; therefore, the final number of subjects included in the analysis was 88 (Tamme et al., 2019a, Tamme et al., 2019b).

The study protocol was approved by the Research Ethics Committee of the University of Tartu (Estonia) (Consent No. 260/T-19, 13 June 2016). Written signed informed consent was obtained from all subjects and additionally from a parent if a participant was younger than 18.0 years old.

The study time points (T1, T2, T3) were defined by the mean age of subjects at the study points: 12.1 years (range 10.6–13.4) at T1, 14.0 years (12.5–15.3) at T2 and 18.0 years (16.5–19.2) at T3 (Table 1). Anthropometry was measured and blood samples were obtained at T1, T2 and T3. Bone age and sexual maturation were studied at T1 and T2. Bone mineral characteristics, body composition and physical activity were studied at T1 and T3.

**Table 1:** Clinical characteristics, blood markers, physical activity data and bone mineral characteristics (DPX-IQ Lunar densitometer at T1 and Discovery Hologic densitometer at T3) of subjects (n = 88) at different time points of the study. Median with 25th and 75th percentile for adipokine data, HOMA-IR and physical activity data and mean ± SD for all other characteristics are shown.

Variable	Time point		
	T1	T2	T3
Clinical characteristics			
Age (years)	12.1 ± 0.7	14.0 ± 0.7⁎	18.0 ± 0.7⁎, †
Body mass (kg)	47.2 ± 12.7	59.61 ± 13.7⁎	73.9 ± 12.1⁎, †
Body height (m)	1.55 ± 0.08	1.69 ± 0.8⁎	1.81 ± 0.07⁎, †
BMI (kg/m^2^)	19.5 ± 4.0	20.60 ± 3.74⁎	22.4 ± 3.3⁎, †
Total body fat %	23.15 ± 10.54		18.07 ± 4.92⁎
Blood markers			
Leptin (ng/mL)	3.40 (1.8; 9.99)	2.3 (0.6; 5.65)⁎	1.85 (0.68; 3.36)⁎, †
Adiponectin (μg/mL)	7.7 (5.0; 11.1)	6.2 (4.5; 9.2)⁎	3.27 (2.64; 4.03)⁎, †
Leptin to adiponectin ratio	0.46 (0.18; 1.37)	0.22 (0.10; 0.94)⁎	0.48 (0.19; 1.03)†
HOMA-IR	1.69 (1.15; 2.68)	2.35 (1.76; 3.24)⁎	1.40 (1.04; 1.84)⁎, †
Testosterone (nmol/L)	4.81 ± 5.65	13.59 ± 6.22⁎	20.23 ± 5.24⁎, †
Physical activity data			
Total PA (counts/min)	434 (359; 573)	350 (283; 497)⁎	380 (303; 498)⁎, †
Bone mineral characteristics			
TB BMD (g/cm^2^)	0.98 ± 0.07		1.23 ± 0.09⁎
LS BMD (g/cm^2^)	0.83 ± 0.09		1.06 ± 0.10⁎
TB LH BMC (g)	1341.7 ± 337.8		2323.04 ± 358.01⁎
LS BMC (g)	27.41 ± 6.77		58.44 ± 9.34⁎
TB BMAD (g/cm^3^)	0.088 ± 0.006		0.095 ± 0.005⁎
LS BMAD (g/cm^3^)	0.147 ± 0.013		0.143 ± 0.013⁎
BMC/height	1110.26 ± 181.62		1590.14 ± 188.91⁎

HOMA-IR, homeostasis model assessment-insulin resistance; PA, physical activity; TB, total body; BMD, bone mineral density; LS, lumbar spine; LH, less head; BMC, bone mineral content; BMAD, bone mineral apparent density.

⁎ Significantly different (P < 0.05) from T1.

† Significantly different (P < 0.05) from T2.

### 2.2 Anthropometry and sexual maturation

Body height (cm) was measured to the nearest 0.1 cm using the Martin metal anthropometer according to the standard technique (GPM anthropological instruments, Zurich, Switzerland). Body mass (kg) was measured to the nearest 0.05 kg using a medical electronic scale (A & D Instruments Ltd., Abington, UK) with the subject wearing light clothes. Body mass index (BMI; kg/m^2^) was calculated as body mass divided by the square of body height.

The pubertal development of the subjects was determined using a self-report questionnaire of pubertal stages according to the Tanner classification method (Marshall and Tanner, 1970), which has been previously validated (Duke et al., 1980). The subjects were given photographs, figures and descriptions representing genitalia and pubic hair development stages and were asked to choose the one that most closely matched their own development. In the case of discrepancies between the two variables, the Tanner stage of the subject was determined according to the self-estimation of genitalia development (Duke et al., 1980). Bone age was determined by the method of Greulich and Pyle using an X-ray of the left hand and wrist (Greulich and Pyle, 1959).

### 2.3 Bone mineral density and body composition

Total body fat percentage and bone mineral characteristics, including total body (TB) BMD (g/cm^2^), lumbar spine (LS; L2-L4) BMD (g/cm^2^), total body less head bone mineral content (TB LH BMC) (g), lumbar spine bone mineral content (LS BMC) and bone area were measured by DEXA scan DPX-IQ (Lunar Corporation, Madison, WI) at T1 and by DEXA scan Discovery (Hologic QDR Series, Waltham, MA, USA) at T3. The subjects were scanned in supine position wearing minimal clothing and medium scan mode was used for measurement. Bone mineral apparent density (BMAD) (g/cm^3^), an estimate of volumetric bone density, was calculated using the formula TB BMAD = TB BMC / (TB bone area^2^ / height) and the formula LS BMAD = LS BMC / bone area^1.5^ (Katzman et al., 1991). In addition, the expression of TB BMC for height (TB BMC/height) was calculated. The precision of measurement expressed as a coefficient of variation (CV) was <2% for all bone mineral measurements.

### 2.4 Blood analyses

Venous blood samples were obtained after an overnight fast between 8:00 AM and 9:00 AM, the blood serum was separated and then frozen at −80 °C for further analysis. Leptin concentration was determined by radioimmunoassay (Mediagnost GmbH, Reutlingen, Germany). This assay had intra- and inter-assay CV of less than 5%, and the least detection limit was 0.01 ng/mL. Adiponectin was determined with a commercially available radioimmunoassay kit (Linco Research, St. Charles, MO). The intra- and inter-assay CV were less than 7%, and the least detection limit was 1 μg/mL. Total testosterone (nmol/L) was determined using Immulite® 2000 (DPC, Los Angeles, USA) with the inter- and intra-assay CVs of less than 5%, and the lowest detection limit was 0.01 nmol/L. Insulin was analysed using Immulite 2000 (DPC Los Angeles, USA). The intra- and inter-assay CVs were less than 5% and 12%, respectively, at an insulin concentration of 6.6 mU/mL. Glucose was measured with a commercial kit (Boehringer, Mannheim, Germany). The estimate of insulin resistance by homeostasis model assessment (HOMA-IR) was calculated: fasting serum insulin (μU/mL) × fasting serum glucose (mmol/L) / 22.5 (Wallace et al., 2004).

### 2.5 Physical activity

Physical activity (PA) was measured objectively by ActiGraph accelerometer (model GT1M (at T1 and T2) and model GT3X (at T3), ActiGraph LLC, Pensacola, FL, USA) designed to register vertical accelerations. All subjects were instructed to wear the accelerometer on the right hip for seven consecutive days during the wake-up time. For the analyses of accelerometer data, all night activity (24:00–6:00 h) and all sequences of 10 min or more of consecutive zero counts were excluded from each individual's recording. At least two weekdays and one weekend day of recording with a minimum of 10 h/day was set as an inclusion criterion. The total PA was expressed as total number of counts divided by the registered time (counts/min).

### 2.6 Statistical analyses

Statistical analyses were performed using SPSS software version 20.0 for Windows (SPSS, Inc., Chicago, IL). All variables were checked for normality of distribution before analysis. Normally distributed continuous variables are described as a mean ± SD and not normally distributed variables as a median and 25th and 75th percentiles. The results of the leptin and adiponectin measurements were log transformed for further analyses. Mean pubertal LAR was calculated using the formula mean pubertal LAR = (LAR at T1 + LAR at T2) / 2. To determine the changes between different time points of the study, a paired *t*-test for normally distributed data and a Mann-Whitney test for not normally distributed data were used. Spearman correlation coefficient was calculated to describe the associations between mean pubertal LAR and bone mineral characteristics at T3. Partial correlation analysis was performed to assess the relationships of bone mineral characteristics with mean pubertal LAR, while total body fat percentage, HOMA-IR, total testosterone and total PA at T1 were included as covariates. P-value of less than 0.05 was considered significant for all analyses.

## 3 Results

The clinical characteristics, biochemical markers and PA data measured at T1, T2 and T3 are outlined in Table 1. The mean age of subjects at the beginning of the study was 12.1 years. Boys at T1 were mainly in pubertal stage 2 (n = 33) and pubertal stage 3 (n = 45) according to the Tanner classification. The mean bone age at T1 was 11.9 ± 1.15 years (95% CI 11.6–12.1) and at T2 13.9 ± 1.1 years (95% CI 13.7–14.2). Median serum leptin and adiponectin concentrations declined significantly over the study period while LAR decreased significantly from T1 to T2 and increased thereafter to T3 (Table 1).

The absolute values of bone mineral characteristics at T1 and T3 are presented in Table 1. Mean pubertal LAR was negatively correlated with LS BMD at T3 (r = −0.23; P < 0.05) and LS BMAD at T3 (r = −0.33; P < 0.05). In partial correlation analysis after controlling for total body fat percentage, total testosterone, HOMA-IR and PA at T1 the correlation between mean pubertal LAR and LS BMD at T3 (r = −0.31; P < 0.05) as well as between pubertal LAR and LS BMAD at T3 (r = −0.41; P < 0.05) remained significant. However, no significant correlations were found between LAR at T3 and the bone mineral characteristics at T3 (results not shown).

## 4 Discussion

The main finding of the present study is that LAR in puberty is negatively associated with LS BMD as well as LS BMAD at the mean age of 18 years in healthy males. These correlations remained significant after adjustment to total body fat percentage, HOMA-IR, total testosterone and total PA at T1.

Adiponectin to leptin ratio (LAR) has been mainly studied in the context of obesity-related disorders such as cardiometabolic diseases (Satoh-Asahara et al., 2004), diabetes (Finucane et al., 2009) and metabolic syndrome (Zhuo et al., 2009). However, as lower bone mass has been found in children with obesity, particularly in adolescence (Dimitri et al., 2012), the alterations in LAR could also contribute to that process (Dimitri et al., 2011). Higher leptin concentration in obese children has been proposed to reduce bone formation relative to resorption and thereby predispose them to lower bone mass and fractures (Dimitri et al., 2011). The effect of leptin on bone metabolism seems to be dose dependent: at lower levels leptin stimulates bone formation, but at higher levels inhibits bone formation (Martin et al., 2007). The proportion of overweight subjects in our study group was relatively modest, only 14.7%. However, at the beginning of the study a large number of subjects (n = 33) were at pubertal stage 2, a stage when boys are known to have peak serum leptin concentration (Clayton et al., 1997). Thus, we hypothesised that the negative association between LAR in puberty and bone mineral characteristics at age the mean age of 18 years could be explained by the negative central action of the relatively high serum leptin concentration on bone remodelling regulation at the crucial time of bone accumulation. This hypothesis is confirmed by the findings from our previous longitudinal study where serum leptin concentration at 12 years of age was inversely associated with the BMC and BMD increment over the next 24 months (Vaitkeviciute et al., 2016b). Leptin was found to be an independent predictor of low BMD at several sites also in a cross-sectional study of Swedish young adult males (Lorentzon et al., 2005).

The role of adiponectin in bone metabolism is also controversial. Laboratory studies in mice overexpressing adiponectin exhibited an increased bone mass accompanied by decreased numbers of osteoclasts (Oshima et al., 2005) whereas adiponectin-deficient mice exhibited a normal bone mass, except for a slight increase of bone mass in certain age groups (William et al., 2009), or the opposite - reduced bone density and cortical bone in adiponectin-knockout mice (Naot et al., 2016). Studies in humans have found both negative (Sayers et al., 2010) as well as positive (Stojanovic et al., 2018; Tamura et al., 2007) relationship between serum adiponectin and bone mineral characteristics. In addition, some data suggest that sex hormones might modulate the effect of adiponectin on bones as negative correlation between adiponectin and BMD was seen only in females, but not in males (Bi et al., 2020).

The median LAR of our subjects at the age of 12, 14 and 18 years was 0.46, 0.22 and 0.48, respectively. According to the cut-offs proposed to assess metabolic risk based on adiponectin to leptin ratio, the ratios of our subjects would be considered normal keeping in mind their inversely proportional relationship (Frühbeck et al., 2018). However, in the context of bone mineral density, no such cut-offs for LAR (or adiponectin to leptin ratio) have been proposed.

We found significant correlations between pubertal LAR and different bone mineral characteristics, but only of lumbar spine region. It is known that the latter describes more trabecular bone whereas total body BMD more cortical bone. Trabecular bone is influenced more by hormonal and metabolic factors, particularly during pubertal development, whereas the main determinant of cortical bone is weight-bearing activity (Mora et al., 1994). This aligns with the findings from our previous study where physical activity was significantly associated with BMD or BMC of total body and femoral neck, but not with BMD or BMC in lumbar spine (Tamme et al., 2019b).

This study has some limitations. At first, different DEXA scanners by different brands were used at T1 and T3. We were not able to carry out in vitro or in vivo cross-calibration between the scanners because the old machine was no longer available at T3 due to technical problems. The second limitation is that bone age and pubertal stage were assessed only at T1 and T2, and the latter by self-assessment. Another limitation was that serum 25(OH)D concentrations as well as calcium and vitamin D intake were not evaluated in the current study. Although our results are limited to a specific group of Caucasian male adolescents, the major strength of the current study is the relatively long study period, including the pubertal period which is known to be critical in regard to bone mineral accumulation.

In conclusion, our findings from this longitudinal study showed a significant negative correlation between LAR in puberty and LS BMD and LS BMAD at 18 years of age in healthy boys. These findings suggest a possible role of adipokines in puberty in further bone mineral accumulation.

## Funding

This work was supported by the 10.13039/501100005992Estonian Ministry of Education and Science Institutional Grant PRG 1120 and PRG 1428.

## CRediT authorship contribution statement

**Reeli Tamme:** Conceptualization, Formal analysis, Investigation, Writing – original draft. **Jaak Jürimäe:** Conceptualization, Methodology, Writing – review & editing, Project administration, Funding acquisition. **Evelin Mäestu:** Formal analysis, Investigation, Data curation, Writing – review & editing. **Liina Remmel:** Investigation, Writing – review & editing. **Priit Purge:** Investigation, Writing – review & editing. **Eva Mengel:** Writing – review & editing. **Vallo Tillmann:** Conceptualization, Methodology, Writing – review & editing, Funding acquisition.

## Declaration of competing interest

The authors declare that they have no known competing financial interests or personal relationships that could have appeared to influence the work reported in this paper.
